# The Impact of Aging on Macroautophagy in the Pre-ovulatory Mouse Oocyte

**DOI:** 10.3389/fcell.2021.691826

**Published:** 2021-06-29

**Authors:** Alexandra E. Peters, Shandelle J. Caban, Eileen A. McLaughlin, Shaun D. Roman, Elizabeth G. Bromfield, Brett Nixon, Jessie M. Sutherland

**Affiliations:** ^1^Priority Research Centre for Reproductive Science, Schools of Biomedical Science & Pharmacy and Environmental & Life Sciences, University of Newcastle, Callaghan, NSW, Australia; ^2^Pregnancy and Reproduction Program, Hunter Medical Research Institute, New Lambton Heights, NSW, Australia; ^3^School of Science, Western Sydney University, Penrith, NSW, Australia; ^4^School of Biological Sciences, Faculty of Science, The University of Auckland, Auckland, New Zealand; ^5^Priority Research Centre for Drug Development, The University of Newcastle, Callaghan, NSW, Australia; ^6^Department of Biochemistry and Cell Biology, Faculty of Veterinary Medicine, Utrecht University, Utrecht, Netherlands

**Keywords:** autophagy, amphisome, lysosome, autophagosome, reproductive system, protein degradation, oocyte quality

## Abstract

Accompanying the precipitous age-related decline in human female fertility is an increase in the proportion of poor-quality oocytes within the ovary. The macroautophagy pathway, an essential protein degradation mechanism responsible for maintaining cell health, has not yet been thoroughly investigated in this phenomenon. The aim of this study was to characterize the macroautophagy pathway in an established mouse model of oocyte aging using in-depth image analysis-based methods and to determine mechanisms that account for the observed changes. Three autophagy pathway markers were selected for assessment of gene and protein expression in this model: Beclin 1; an initiator of autophagosome formation, Microtubule-associated protein 1 light chain 3B; a constituent of the autophagosome membrane, and lysosomal-associated membrane protein 1; a constituent of the lysosome membrane. Through quantitative image analysis of immunolabeled oocytes, this study revealed impairment of the macroautophagy pathway in the aged oocyte with an attenuation of both autophagosome and lysosome number. Additionally, an accumulation of amphisomes greater than 10 μm^2^ in area were observed in aging oocytes, and this accumulation was mimicked in oocytes treated with lysosomal inhibitor chloroquine. Overall, these findings implicate lysosomal dysfunction as a prominent mechanism by which these age-related changes may occur and highlight the importance of macroautophagy in maintaining mouse pre-ovulatory oocyte quality. This provides a basis for further investigation of dysfunctional autophagy in poor oocyte quality and for the development of therapeutic or preventative strategies to aid in the maintenance of pre-ovulatory oocyte health.

## Introduction

Infertility is a major health burden affecting an estimated 15% of couples globally, with a female factor implicated in approximately half of all cases (Vayena et al., 2002; Agarwal et al., 2005; Vander Borght and Wyns, 2018). Notably, female fertility decreases significantly after the age of 35 (Spira, 1988; te Velde and Pearson, 2002; Leridon, 2004), creating a restricted fertility window that is dictated by the number and quality of oocytes within the ovarian reserve (te Velde and Pearson, 2002; Doherty and Pal, 2011). During mammalian embryonic development, all oocytes enter meiosis and arrest in prophase I until selected for ovulation some decades later (Hsueh et al., 2015). This prolonged stage of cell cycle arrest renders the oocyte vulnerable to damage from a variety of insults such as the oxidative stress and inflammation associated with folliculogenesis and ovulation (Chao et al., 2005; Goud et al., 2008; Miyamoto et al., 2010; Lliberos et al., 2021). Chronic exposure to these factors throughout a female’s reproductive life can cause damage to the intracellular environment of the oocyte and its immediate supportive cells, contributing to reduced oocyte quality. Evidence of this well-established phenomenon is apparent in the elevated aneuploidy rates of oocytes retrieved from women^1^ over 35; where at least 50% of the oocytes in women aged >40 are rendered non-viable (Kuliev et al., 2003; De Bruin and Te Velde, 2004; Fu et al., 2014). Cumulatively, the limiting nature of female fertility results in age-related infertility being the single largest cause of human infertility (Doherty and Pal, 2011; Agarwal et al., 2015). Despite various pathways’ involvement in oocyte aging, there are minimal therapeutic or preventative treatments available to maintain or improve oocyte quality or enhance the success of interventions such as assisted reproductive technologies (Newman et al., 2019; Peters et al., 2020). This situation provides a clear imperative for further investigation into the molecular pathways that underpin female age-related infertility.

One such pathway that warrants consideration in the context of oocyte aging is autophagy; a ubiquitous protein degradation pathway that plays a critical role in cellular protein homeostasis (proteostasis). Autophagy pathways can be activated in response to a diverse range of stressors such as oxidative stress, hypoxia, thermal fluctuations, and nutrient deprivation (Levine and Kroemer, 2008). All autophagy pathways utilize the lysosome, an acidic intracellular vesicle containing hydrolytic enzymes to facilitate the breakdown of aged, damaged, and dysfunctional proteins and organelles (Feng et al., 2014; Klionsky et al., 2016). Importantly, somatic cell studies have demonstrated that the efficiency of autophagy pathways decreases during aging, particularly in long-lived cells such as neurons and cardiac myocytes. This decreased efficiency results from attenuation of lysosomal degradation capacity combined with reduced production, fusion, and elimination of autophagosomes (Terman, 1995; Terman and Brunk, 2004; Essick and Sam, 2010).

**FIGURE 1:**
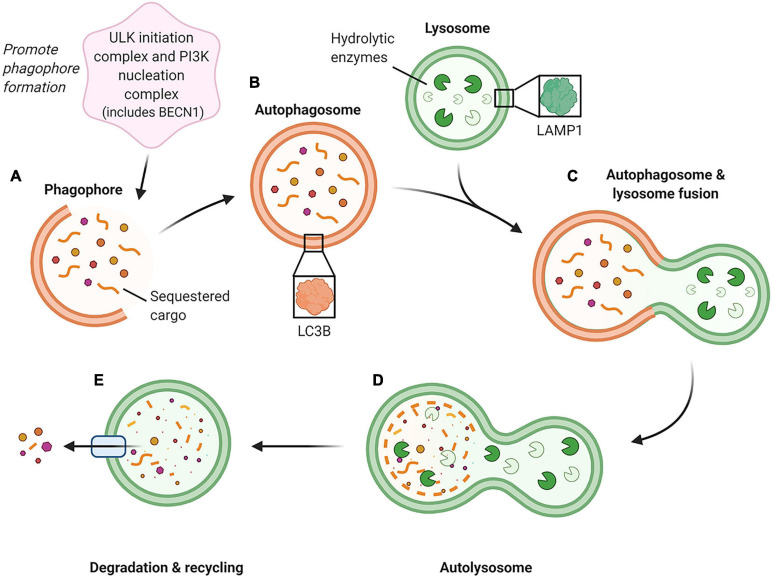
Schematic of the macroautophagy pathway. This pathway is initiated by two groups of proteins, the ULK (unc-51-like kinase 1) initiation complex and the PI3K (phosphoinositide 3-kinase) nucleation complex containing BECN1, which generate the phagophore (immature autophagosome) **(A)**. As the phagophore continues to grow and sequester cargo, it forms a completed double-membraned vesicle termed the autophagosome, which encompasses the cargo to be degraded **(B)**. The autophagosome then proceeds to fuse with an acidic lysosome containing hydrolytic enzymes **(C)** to form an autolysosome. **(D)** Within the autolysosome, the encapsulated cargo is broken down into its basic building blocks and is then extruded back into the cell cytoplasm for recycling **(E)**. (Feng et al., 2014; Klionsky et al., 2016) Created with BioRender.com.

Captured under the umbrella of autophagy are numerous degradation pathways responsible for targeting different cellular components. However, the autophagy pathway with the most diverse and best-characterized roles is macroautophagy (Klionsky and Codogno, 2013). The macroautophagy pathway employs the autophagosome, a double membraned intracellular vesicle to sequester and deliver cargo to the lysosome (Seglen et al., 1990; Feng et al., 2014) (Figure 1). This cargo, usually consisting of aged or damaged proteins, is destined for hydrolysis into its core amino acid building blocks, which are then recycled for use within cellular biosynthetic pathways (Seglen et al., 1990; Feng et al., 2014). Among the key macroautophagy protein components are Beclin-1 (BECN1; involved in the promotion of autophagosome formation), microtubule associated protein 1 light chain 3B (LC3B; an essential constituent of the autophagosome membrane), and lysosomal associated membrane protein 1 (LAMP1; a primary constituent of the lysosome membrane) (Feng et al., 2014; Klionsky et al., 2016) (Figure 1).

Within the ovarian environment, there is evidence that macroautophagy is essential for promoting oocyte survival and development. By way of illustration, the knockdown of *Becn1* in porcine oocytes leads to a reduction in both BECN1 and LC3B protein expression and promotes a suite of phenotypic responses. These responses include reduced polar body extrusion during meiosis, increased DNA damage, and impaired mitochondrial function; changes that ultimately compromise the developmental potential of the oocyte (Shen et al., 2018). Comparable effects have also been documented in response to the use of an upstream inhibitor of autophagy, LY294002 (Shen et al., 2018). Furthermore, the selective knockout of *Becn1* or autophagy-related gene, *Atg7*, significantly reduced or eliminated the oocyte pool within the ovaries of the *Becn1* and *Atg7* deficient mice, respectively (Gawriluk et al., 2011). It should be noted that autophagy can fulfill dual roles in the ovary, whereby it is not only responsible for promoting oocyte survival but alternatively, can promote cell death. Indeed, autophagy pathways operating alongside other cell death mechanisms, such as apoptosis and necroptosis, have been implicated in follicular atresia, an important mechanism responsible for oocyte loss with increasing age (Escobar et al., 2008; Yadav et al., 2018; Chaudhary et al., 2019).

In addition to promoting oocyte survival and growth, autophagy activation has been detected in response to various stress-inducing stimuli. For instance, elevated levels of GFP-LC3 puncta, indicative of autophagy pathway activation, have been detected in mouse oocytes subjected to cold stress (Bang et al., 2014). Activation of autophagy has also been observed in bovine oocytes in response to heat shock (Latorraca et al., 2020). Similarly, the ovulated oocytes of obese mice present with increased levels of autophagy accompanied by mitochondrial dysfunction compared to those harvested from lean mice (Wu et al., 2015). Finally, exposure of female mice to the dietary toxicant acrylamide has been shown to increase the expression of autophagy-related genes such as *Lc3* (a biomarker of autophagy activation) within the ovaries of treated animals (Aldawood et al., 2020). Taken together, such studies allude to the demonstrable importance of autophagy in maintaining oocyte health. However, the basis by which autophagy operates on a subcellular level in the oocyte and its alteration during reproductive aging remains largely unexplored. To address this knowledge gap, this study aimed to characterize components of the macroautophagy pathway in preovulatory oocytes and assess how the fidelity of this pathway is impacted during natural maternal aging.

## Materials and Methods

### Animal Ethics

Research animals in this study were handled, monitored, and euthanized in accordance with NSW Animal Research Act 1998, NSW Animal Research Regulation 2010 and the Australian Code for the Care and Use of Animals for Scientific Purposes 8th Ed. as approved by the University of Newcastle Animal Care and Ethics Committee (approval number A-2018-803). C57/BL6 × CBA hybrid (F1) mice were bred by the University of Newcastle Animal Services Unit and housed under a 12 h light/12 h dark cycle at a constant temperature of 21–22°C with food and water supplied *ad libitum*. Mice were euthanized immediately before oocyte collection *via* carbon dioxide inhalation, and death was confirmed through subsequent cervical dislocation.

C57/BL6 × CBA hybrid (F1) mice were utilized at two critical time points: 4–6 weeks old, equivalent to a human with initial fertility, and 12–14 months old, equivalent to a human with declining fertility. These time points were selected based on an established model of oocyte aging in our research group that exhibits similar age-related changes in oocyte quality and quantity as humans (Camlin et al., 2017b).

### Oocyte Collection

Unstimulated ovaries were removed from euthanized mice aged between 4 and 6 weeks (young time point) or 12–14 months of age (aged time point). Ovaries were dissociated by repeatedly puncturing pre-ovulatory follicles with a 27-gauge needle to release mature pre-ovulatory oocytes as cumulus-oocyte complexes into pre-warmed (37°C) M2 media (Merck, Darmstadt, Germany) supplemented with 2.5 μM milrinone to maintain prophase I arrest. Media droplets were kept under embryo tested mineral oil (Merck) in 35 mm Petri dishes (Cellstar, Baltimore, MD, United States). Cumulus cells were mechanically removed *via* repeated aspiration with a narrow pipette and washed through M2 media droplets until no cumulus cells or debris remained.

### RNA Extraction, Reverse Transcription, and Quantitative PCR (RT-qPCR)

Total RNA was extracted from 40 oocytes per replicate following removal of the zona pellucida *via* incubation in acid Tyrode’s solution (Merck). RNA was subsequently snap-frozen in liquid nitrogen and stored at −80°C. Reverse transcription was performed to generate cDNA using a TaqMan Gene Expression Cells-to-CT kit (Thermo Fisher Scientific, Waltham, MA, United States) as per the manufacturer’s instructions and stored at −80°C.

Quantitative real-time PCR (RT-qPCR) was performed on cDNA using TaqMan assay probes and TaqMan gene expression master mix (Thermo Fisher Scientific) according to the manufacturer’s instructions. The assay probes used were *Becn1* (assay ID. Hs01007018_m1), *Map1lc3* (assay ID. Mm00782868_sH), and *Lamp1* (assay ID. Mm00495262_m1). RT-qPCR for each biological replicate was performed in triplicate using 2 μl of cDNA for 40 amplification cycles (50°C pre-incubation for 120 s, 95°C pre-incubation for 600 s, followed by 40 cycles of 95°C for 15 s and 60°C for 60 s) with a LightCycler 96 (Roche Diagnostics, Basel, Switzerland). For each sample, a replicate omitting the reverse transcriptase enzyme was used as a negative control. All data were analyzed using LightCycler 96 software (Version 1.1) according to the equations of Schmittgen and Livak (2008). Data were normalized to the housekeeping gene, peptidylprolyl isomerase A (*Ppia*) (assay ID. Mm02342429_g1), and then presented relative to young oocytes where appropriate. *Ppia* was chosen as an appropriate control due to its previous use in oocytes, its maintained expression levels between groups, and detection at earlier cycle values than that of the genes of interest (Mihalas et al., 2019).

### SDS-PAGE and Immunoblotting

Protein was extracted from oocytes by incubating in NuPAGE 4 × lithium dodecyl sulfate (LDS) sample buffer (Invitrogen, Waltham, MA, United States) diluted in RIPA buffer (150 mM NaCl, 50 mM Tris pH 8, 0.1% w/v SDS, 0.5% w/v sodium deoxycholate, 1% v/v Triton X-100) supplemented with protease/phosphatase inhibitor cocktail (Thermo Fisher Scientific) and 4% v/v β-mercaptoethanol (Merck) at 100°C for 10 min. Protein extracts equivalent to 50 oocytes per lane were loaded into a 12% Bis-Tris Bolt pre-cast gel (Invitrogen), and electrophoresis was performed at a constant voltage of 130 V. This was followed by western transfer onto a 0.45 μm PVDF membrane (GE Healthcare, Amersham, Buckinghamshire, United Kingdom) at a constant current of 350 mA for 90 min. Membranes were then blocked with 5% w/v skim milk diluted in Tris-buffered saline (TBS) with 1% v/v Tween-20 (TBST) for 2 h at room temperature. Following blocking, membranes were incubated with primary antibodies specific for BECN1, LC3B, or LAMP1 diluted as in Supplementary Table 1 in 1% w/v skim milk-TBST at 4°C overnight. Membranes were washed 3 times (2 × 10 min followed by 1 × 30 min) in TBST and then incubated with horseradish peroxidase-conjugated secondary antibody diluted in 1% w/v skim milk-TBST for 2 h at room temperature. Blots were washed 3 times (2 × 10 min followed by 1 × 30 min) again in TBST prior to being developed using an enhanced chemiluminescence kit (GE Healthcare). After development, membranes were stripped using Western ReProbe (G-Biosciences, Maryland Heights, MO, United States) and re-probed with anti-glyceraldehyde 3-phosphate dehydrogenase (GAPDH) antibodies as in Supplementary Table 1 in TBST. Densitometry was performed using Amersham Imager 600 software (GE Healthcare), and protein expression of target proteins was normalized against GAPDH loading control.

### Immunocytochemistry

Following oocyte collection, live cells were rinsed 3 times in phosphate-buffered saline (PBS) containing 3 mg/ml polyvinylpyrrolidone (PVP). They were then fixed and permeabilized in 2% v/v paraformaldehyde containing 0.5% v/v Triton-X for 30 min. Fixed oocytes were rinsed 3 times again in PBS/PVP before being blocked with 7% v/v normal goat serum (Merck) in 1% w/v BSA with 0.1% v/v Tween-20 in PBS (PBST) for 1 h at room temperature. The oocytes were then incubated with primary antibodies specific for BECN1, LC3B, LAMP1, or EEA1 diluted as in Supplementary Table 1 in 1% w/v BSA-PBST overnight at 4°C. Oocytes were washed 3 times (2 × 5 min followed by 1 × 60 min) in 1% w/v BSA-PBST before being incubated with relevant Alexa Fluor-conjugated secondary antibodies (Thermo Fisher Scientific) diluted 1:500 in 1% w/v BSA-PBST for 1 h at room temperature. All experiments included secondary antibody only controls in which the primary antibody was substituted with antibody/wash buffer (Supplementary Figure 1). Oocytes were once again washed three times (2 × 5 min followed by 1 × 60 min) in 1% w/v BSA-PBST and counterstained with 4′, 6-diamidino-2-phenylindole (DAPI) diluted 1:5000 in PBS for 15 min at room temperature. Oocytes were mounted onto 12 well slides (Thermo Fisher Scientific) in 1 μL of Citifluor Glycerol Solution AF2 (Citifluor Ltd., London, United Kingdom) per well. Pre-ovulatory oocytes with a chromatin formation indicative of reduced capacity to complete embryonic development (non-surrounded nucleus) (Christians et al., 1999) were excluded from each group upon assessing the chromatin arrangement stained with DAPI.

### Confocal Imaging

Oocyte images were captured via confocal microscopy on an Olympus FV1000 confocal microscope under a 60× oil immersion lens. For each oocyte, a *z*-stack was performed with 1 μm intervals for 15 μm total through the center of the oocyte capturing the whole nucleolus. Different color channels were imaged sequentially to avoid bleed-through, and a Kalman filter was applied to reduce background fluorescence. Excitation lasers used were 405 nm (DAPI), 559 nm (Alexa Fluor 555), and 635 nm (Alexa Fluor 633) with the emission imaged for analysis. In each group, 5–15 mature oocytes were imaged for analysis.

### Puncta Analysis: Measurement of Puncta Per Cell

**FIGURE 2:**
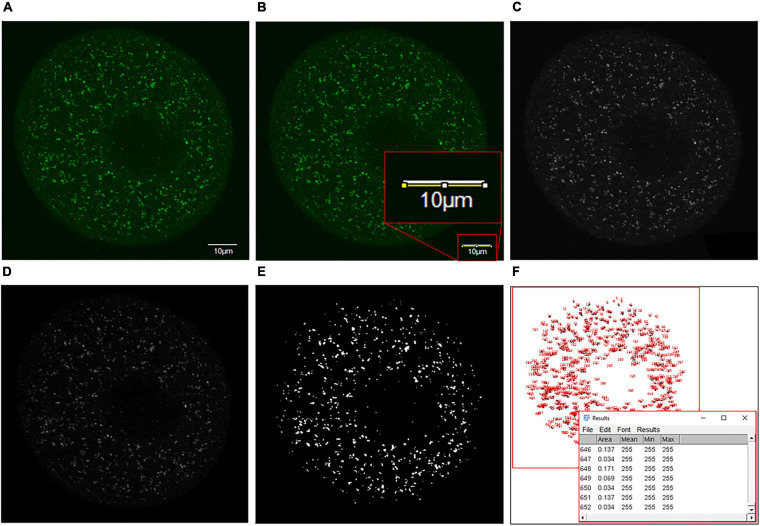
Details of the puncta analysis method utilized to analyze puncta size distribution within immunolabeled oocyte images. **(A)** Firstly, the single-channel image of antibody staining to be analyzed with a scale bar of known size was acquired. **(B)** The scale bar was traced using the straight-line tool, and the scale of the image was adjusted from pixels to microns (Analyze > Set Scale > Input scale bar size as known distance). The scale bar was then circled and deleted using an area selection tool to remove from the image. **(C)** The image was converted to an 8-bit grayscale format for further analysis (Image > Type > 8-bit). **(D)** White top hat transformation (Plugins > Morpholibj > Morphological filters) was then performed. To do this, the ‘White top hat’ operation was selected, and parameters were adjusted to obtain a reduced background and enhance features of interest. **(E)** The image was then thresholded (Image > Adjust > Threshold) by selecting the threshold that most accurately represented the detail of the initial image (*T* = 20 here). **(F)** Finally, the ‘analyze particles’ feature was selected (Analyze > Analyze particles) to produce a dialog box containing the number and size of each puncta measured. This information was copied into Excel, where puncta were sorted by size and categorized as desired.

Firstly, slices of each 15 μm *z*-stack were overlaid into a single image for puncta analysis using Fiji software (extension of ImageJ version 1.53c) (Schindelin et al., 2012). For each image, the unit of measurement was converted from pixels to microns using the scale bar, followed by converting the image to an 8-bit format. The MorphoLibJ plugin (Legland et al., 2016) was then used, with the morphological filter ‘White Top Hat’ to reduce background fluorescence that can obscure puncta size (Qvarnström et al., 2004). Next, the images underwent thresholding to isolate the puncta visible in the original image (Figure 2). Thresholds were kept consistent across experimental groups within each replicate. Finally, the number of puncta was assessed, producing a numbered list of each puncta and its associated area in μm^2^. For each oocyte, the output for the number of puncta was sorted by size in Microsoft Excel, and the total size distribution was compared between groups in GraphPad Prism 8.4.3 (San Diego, CA, United States). Size categories assessed were defined by the relevant vesicle labeled by each marker along with additional size categories for other puncta detected to assess all potential differences within each dataset. These size categories included the recorded autophagosome size (Mizushima et al., 2002) (0.5–1.5 μm in diameter or 0.196–1.767 μm^2^ in area) for LC3B and recorded lysosome size (Ponsford et al., 2020; Trivedi et al., 2020) (0.03–0.5 μm^2^ in area) for LAMP1. Additional puncta detected outside of these categories were assessed in arbitrary categories increasing by 1 μm^2^ up to >10 μm^2^ for LC3B and BECN1, and increasing by 0.1 μm^2^ up to >1 μm^2^ for LAMP1. The differences between experimental groups for each category were then determined through statistical analysis as detailed below.

### Puncta Analysis: Periphery Measurements

The proportion of LC3B and BECN1 puncta greater than 10 μm^2^ residing at the cell’s periphery was also measured. This measurement was carried out using the same process as above (puncta analysis: measurement of puncta per cell) with an extra step to select for the outer 10 μm of the cell. The number of puncta greater than 10 μm^2^ in the periphery of each cell was then compared between young and aged groups, and differences were determined via statistical analysis as detailed below.

### Colocalization Analysis

Using Fiji software (an extension of ImageJ version 1.53c), each two-channel image was split into its individual channels, and the morphological top-hat filter was applied (MorphoLibJ plugin). The Coloc 2 plugin was then used to produce an output displaying several statistical colocalization tests showing the relationship between the localization of the two different channels. From these data, Pearson’s correlation coefficients (no threshold) were obtained as indicators of colocalization of the target proteins within each oocyte, and groups were then compared.

### Proximity Ligation Assay

Proximity ligation assays (PLA) were performed on fixed oocytes using a Duolink *In Situ* Red Starter kit (Merck) as per the manufacturer’s instructions with slight modification. Briefly, oocytes were blocked with Duolink blocking solution for 1 h at 37°C and then incubated with primary antibodies LC3B and EEA1 diluted as in Supplementary Table 1 in Duolink antibody buffer overnight at 4°C. Labeled oocytes were then washed with PBS/PVP supplemented with 1% v/v Tween-20 (3 × 10 min) before incubation with oligonucleotide-conjugated secondary antibodies (positive and negative PLA probes) for 1 h at 37°C. After additional washes (3 × 10 min), the ligation and amplification of PLA probes were conducted according to the manufacturer’s instructions. Finally, oocytes were counterstained with DAPI at a 1:5,000 dilution in PBS for 15 min at room temperature. Positive control incubations included antibody pairs targeting proteins expected to interact, including α-tubulin and β-tubulin (Supplementary Table 1). Negative control incubations used to confirm the specificity of the assay included antibody pairs targeting proteins that would not be expected to interact; LC3B and PIWIL1 (Supplementary Table 1), as well as the omission of primary antibodies (Supplementary Figure 2).

### Chloroquine Treatment

Titration of effective doses of chloroquine phosphate (Abcam) was first performed using working solutions of between 0 and 400 μM chloroquine prepared in M2 media (Merck) supplemented with 2.5 μM milrinone to maintain prophase I arrest. Live oocytes were washed through 2 × 100 μL droplets of each relevant treatment before incubation in a third 100 μL droplet under mineral oil (Merck) in a 35 mm embryo tested Petri dish (Cellstar) for 6 h at 37°C in the dark. Oocytes were then fixed as previously described, and immunocytochemistry was performed to assess anti-LAMP1 antibody labeling (Supplementary Table 1). Analysis was performed in the same manner as previously described, focusing on assessing lysosomal dilation using LAMP1. Lysosomal dilation indicated chloroquine treatment had effectively prevented lysosomal fusion with autophagosomes and inhibited autophagy (Mauthe et al., 2018). Oocyte viability was monitored visually post-treatment on live oocytes, and any other irregularities in morphology were captured and noted during imaging of fixed cells. Based on the results from initial dose-response experiments, a concentration of 200 μM chloroquine was selected as the optimal treatment. This concentration was used to assess the impact of lysosomal inhibition on LC3B and EEA1 (Supplementary Table 1) immunolabeling within oocytes.

### Statistical Analyses

All data were normalized to the young or control group and analyzed using a two-tailed unpaired Student’s *t*-test or one-way ANOVA followed by a multiple comparisons test in GraphPad Prism 8.4.3. All experiments were performed with at least 3 biological replicates (specified for each set of experiments in results). Unless otherwise stated, all data are presented as mean ± standard error of the mean (SEM). Statistical significance was considered as ^∗^*P* < 0.05, or ^∗∗^*P* ≤ 0.01.

## Results

The initial goal of this study was to characterize the macroautophagy pathway in mouse pre-ovulatory oocytes. These oocytes were retrieved from young (4–6 week-old, equivalent to a human with initial fertility) and aged (12–14 month-old, equivalent to a human with declining fertility) mice. These age groups, from now on referred to as ‘young’ and ‘aged,’ were selected based on a pre-established model of oocyte aging within our laboratory. This model demonstrates decreased oocyte quantity and quality comparable to humans at their corresponding age time points (Camlin et al., 2017b).

### Relative Expression of Macroautophagy Marker Genes *Map1lc3*, *Becn1*, and *Lamp1* Is Not Altered in Aged Mouse Oocytes

**FIGURE 3:**
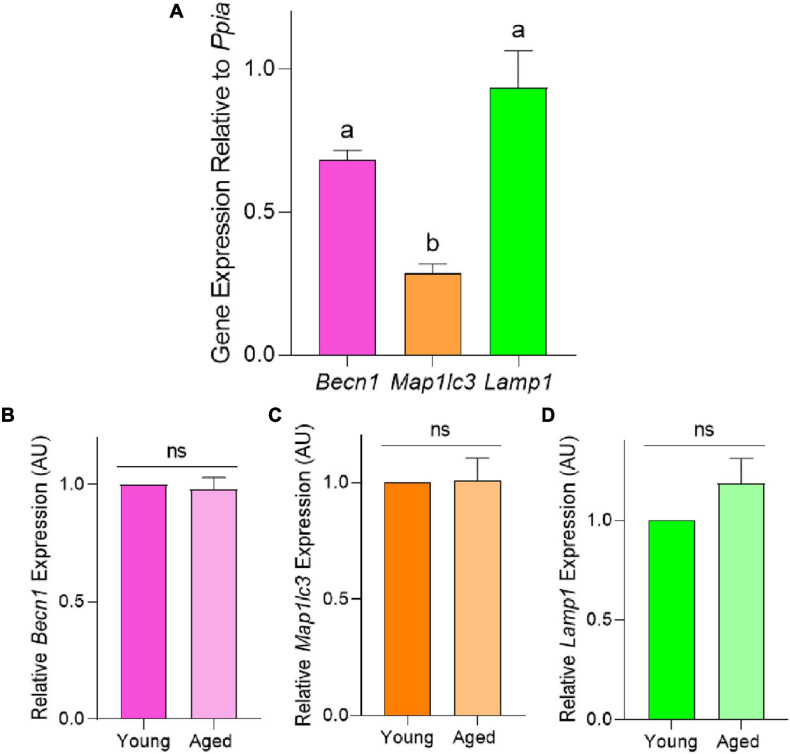
Relative mRNA expression of autophagy pathway members *Becn1*, *Map1lc3*, and *Lamp1* in the pre-ovulatory oocytes of young and aged mice. RT-qPCR was performed on oocytes collected from young (4–6 weeks old) and aged (12–14 months old) mice to assess the target gene expression relative to *Ppia*, the steady state housekeeping gene. **(A)** Comparison of *Becn1*, *Map1lc3*, and *Lamp1* expression in young oocytes. Comparison of relative **(B)** *Becn1*, **(C)** *Map1lc3*, and **(D)** *Lamp1* gene expression in populations of young and aged mouse oocytes (normalized to young). All data are presented as mean ± SEM (*n* = 4), with statistical significance (*P* < 0.05) between target gene expression in panel **(A)** denoted by different letters; ns, not significant.

To characterize the macroautophagy pathway in mouse pre-ovulatory oocytes, we initially focused on assessing the expression of the recognized autophagy markers *Becn1*, *Map1lc3*, and *Lamp1*. The expression of these markers was assessed in pre-ovulatory oocytes retrieved from young mice via RT-qPCR and normalized relative to the steady-state housekeeping gene, *Ppia* (Figure 3A). This strategy successfully amplified transcripts for each of the three gene targets, with *Lamp1* proving to be the most highly expressed, followed by *Becn1* and *Map1lc3.* Notably, the relative expression of *Map1lc3* proved significantly lower than *Lamp1* (*p* = 0.0006) and *Becn1* (*p* = 0.0148). In extending this analysis to focus on the expression of *Becn1*, *Map1lc3*, and *Lamp1* genes in aged oocytes, there were no significant age-related differences in target transcript abundance (Figures 3B–D).

### Macroautophagy Related Proteins BECN1, LC3B-II, and LAMP1 Are Expressed at Equivalent Levels in Young and Aged Mouse Oocytes

**FIGURE 4:**
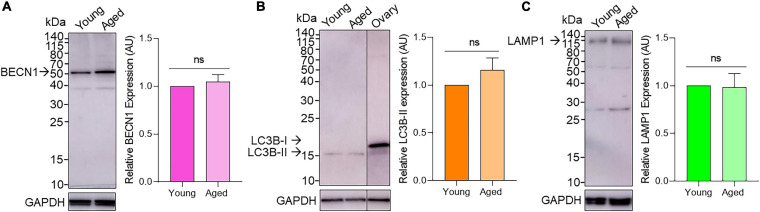
Assessment of BECN1, LC3B, and LAMP1 protein expression in young and aged pre-ovulatory mouse oocytes. Oocyte protein lysates from young (4–6 weeks old) and aged (12–14 months old) mice were subjected to immunoblotting with **(A)** anti-BECN1 (52kDa), **(B)** anti-LC3B (19 and 17 kDa for LC3B-I and LC3B-II, respectively; including whole ovary positive control) or **(C)** anti-LAMP1 (120kDa) antibodies. Following detection of the target antigens, the blots were stripped and reprobed for the housekeeping protein GAPDH (35 kDa) to ensure equivalent protein loading in each lane. Densitometric analysis was performed on labeled bands corresponding to the predicted molecular weight of target proteins (please see arrows) to determine the protein abundance relative to GAPDH. Densitometric data are expressed as mean ± SEM (*n* = 4, normalized to young) with statistical analysis revealing no significant (ns) differences in target protein expression between young and aged oocytes.

Immunoblotting confirmed the expression of BECN1, LC3B, and LAMP1 proteins in mouse oocytes. As shown in Figure 4, the three target proteins were readily detected in both young and aged populations of oocytes with minor non-specific cross-reactivity observed. Densitometric analysis of target band intensity revealed no significant differences in the abundance of BECN1, LC3B, or LAMP1 proteins in young and aged oocytes (Figure 4 and Supplementary Figure 3). Despite the detection of two bands denoting the presence of LC3B-I (19 kDa) and LC3B-II (17 kDa) in whole ovarian protein, only the band corresponding to LC3B-II was identified in isolated oocytes (Figure 4B).

### BECN1 Immunolocalization Is Unchanged in Young and Aged Mouse Oocytes

Having confirmed the presence of BECN1, LC3B, and LAMP1 in mouse oocytes, we next assessed the localization of these three proteins within oocytes using standard immunocytochemistry labeling techniques. Immunocytochemistry was accompanied by a specific image analysis method designed for this study to measure the number and area of all stained puncta captured within the cell. Standardization for this method was achieved by capturing *z*-stacked images through the middle 15 μm of each oocyte (please refer to section “Materials and Methods”). This strategy allowed the assessment of subcellular localization and generated quantitative data of the number, size, and distribution of labeled vesicles within the sampled cell. It should be noted that quantitative data of puncta numbers does not necessarily reflect absolute values; however, this strategy allowed for comparative assessment of puncta numbers between groups. The details of which can yield important insight into the mechanics of the macroautophagy pathway.

**FIGURE 5:**
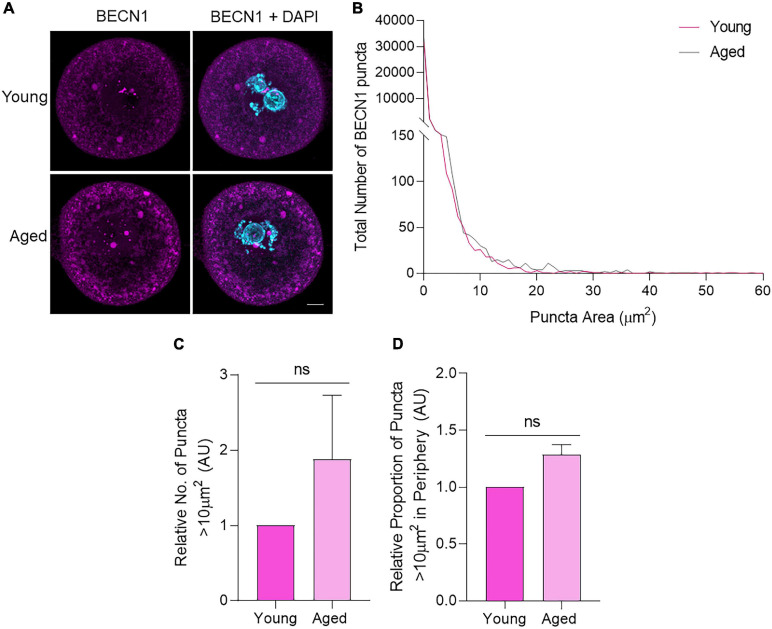
BECN1 protein localization and puncta distribution in young and aged mouse oocytes. Oocytes from young (4–6 weeks old) and aged (12–14 months old) mice were fixed prior to labeling with anti-BECN1 antibodies (pink), nuclear counterstaining with DAPI (cyan), confocal imaging (60× objective), and puncta analysis. **(A)** Representative images are presented to illustrate the punctate cytoplasmic and nuclear localization of BECN1 in young and aged oocytes. Scale bar = 10 μm. **(B)** Images were assessed for the number and area of BECN1 labeled puncta, and these data were used to graph the size distribution of puncta in populations of young (pink line) and aged oocytes (gray line). **(C)** Comparison of the number of BECN1 labeled puncta >10 μm^2^ in young and aged oocytes (normalized to young). **(D)** Comparison of the proportion of BECN1 labeled puncta >10 μm^2^ that resided within the periphery (outermost 10 μm) of the oocyte (normalized to young). *N* = 3, ns, not significant.

Focusing first on BECN1, immunocytochemistry of young and aged oocytes revealed a distinct punctate distribution of this protein throughout the oocyte cytoplasm and nuclear domain (Figure 5A). Notably, the size distribution profile of the BECN1 labeled puncta proved to be highly variable within both young and aged oocytes. In this context, 98% of puncta ranged between 0 and 2 μm^2^. In assessing puncta >2 μm^2^, we noted a reciprocal relationship whereby the larger the puncta area, the fewer the number of puncta detected in that size range (Figure 5B). Despite this variability, we did not record any significant changes in puncta size profiles between the young and aged oocyte groups. Notable trends in our data included an increased number of large puncta greater than 10 μm^2^ in aged oocytes, accompanied by the accumulation of these puncta toward the periphery of the aged cells (Figures 5C,D). The latter was assessed by calculating the percentage of puncta residing in the outer 10 μm diameter of the oocyte cytoplasm.

### Accumulation of Large LC3B Labeled Puncta in Aged Mouse Oocytes

**FIGURE 6:**
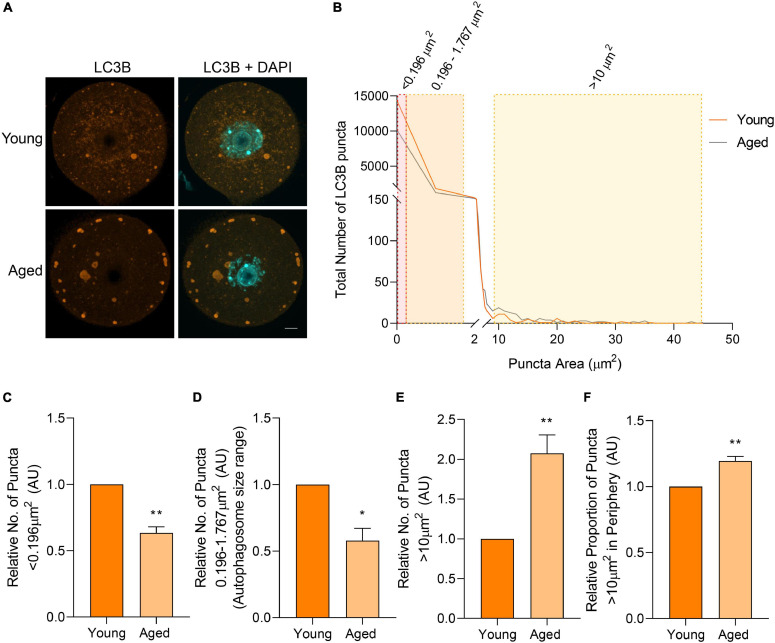
LC3B protein localization and puncta distribution in young and aged mouse oocytes. Oocytes from young (4–6 weeks old) and aged (12–14 months old) mice were fixed prior to labeling with anti-LC3B antibodies (orange), nuclear counterstaining with DAPI (cyan), confocal imaging (60× objective), and puncta analysis. **(A)** Representative images illustrate the punctate cytoplasmic localization of LC3B in young and aged oocytes. Scale bar = 10 μm. **(B)** Images were assessed for the number and size of LC3B labeled puncta, and these data were used to graph the size distribution of puncta in populations of young (orange line) and aged oocytes (gray line). The puncta size groupings that differed significantly in abundance between young and aged oocytes are highlighted in red, orange, and yellow shading. **(C)** Comparison of the number of LC3B labeled puncta <0.196 μm^2^, **(D)** 0.196–1.767 μm^2^ [documented area of autophagosome (Mizushima et al., 2002)], **(E)** and >10 μm^2^ in young and aged oocytes (normalized to young). **(F)** Comparison of the proportion of LC3B labeled puncta >10 μm^2^ that resided in the periphery (outermost 10 μm) of the oocyte (normalized to young). *N* = 3, *P < 0.05, **P ≤ 0.01.

Immunocytochemical labeling of oocytes with anti-LC3B antibodies revealed a similar localization profile to BECN1, with punctate staining distributed throughout the cell cytoplasm (Figure 6A). Similarly, the size distribution of LC3B labeled puncta demonstrated a comparable trend to that of BECN1, with a large proportion of the puncta identified between 0 and 2 μm^2^. Once again, the proportion of LC3B labeled puncta decreased as their area increased. Unlike BECN1, however, significant differences were observed in the distribution of LC3B puncta across several size categories when comparing young and aged oocytes (Figure 6B). The specific size categories of interest included those less than 0.196 μm^2^, distributed between 0.196 and 1.767 μm^2^, and those greater than 10 μm^2^ (Figure 6B; shaded areas). These categories pertain to the predicted sizes of specific biological structures that contain LC3B. Puncta within the category of less than 0.196 μm^2^ are likely to correspond to phagophores, immature double-membrane structures that grow to form autophagosomes. This particular size group experienced a significant age-related reduction (*p* = 0.001) in the older oocyte population (Figure 6C). The next size category, 0.196–1.767 μm^2^, corresponds to the documented size of autophagosomes [0.5–1.5 μm in diameter (Mizushima et al., 2002)], and also displayed a significantly decreased number of puncta (*p* = 0.011) in aged oocytes (Figure 6D). Conversely, aged oocytes were characterized by a significant accumulation (*p* = 0.009) of puncta greater than 10 μm^2^. Moreover, these larger puncta were significantly more likely to reside within the periphery (outermost 10 μm) of the aged oocyte cytoplasm (*p* = 0.005) (Figures 6E,F). This differential localization and large size compared to that of a typical autophagosome prompted a more detailed characterization of the identity of this latter class of LC3B labeled puncta (as described below).

### Large LC3B Positive Puncta Identified as Amphisomes Containing EEA1 in Mouse Oocytes

**FIGURE 7:**
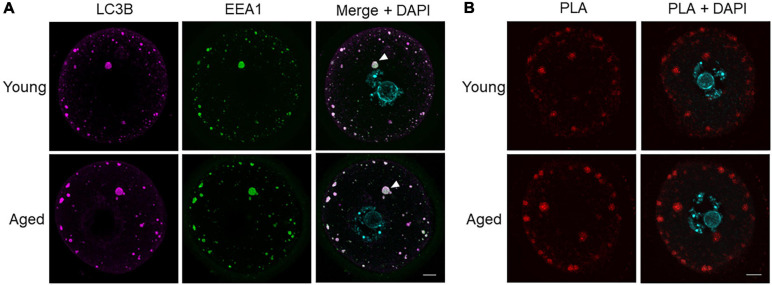
Co-localization and proximity ligation assay for LC3B and EEA1 demonstrating presence of amphisomes in young and aged mouse oocytes. **(A)** Oocytes from young (4–6 weeks old) and aged (12–14 months old) mice were fixed prior to labeling with anti-LC3B antibodies (pink), anti-EEA1 antibodies (green), nuclear counterstaining with DAPI (cyan), confocal imaging (60× objective), and puncta analysis. Representative images illustrate the punctate cytoplasmic co-localization of LC3B and EEA1 in young and aged oocytes where white arrow points to an example of large puncta. Scale bar = 10 μm, *N* = 4. **(B)** Fixed young and aged oocytes underwent a proximity ligation assay for LC3B and EEA1 and were imaged through confocal microscopy (60× objective). Representative images illustrate the characteristic punctate staining similar to that in **(A)** where red signal indicates proteins are within 40 nm.

We next sought to determine the identity of LC3B-staining puncta greater than 10 μm^2^ and further characterize the changes observed in these puncta between aged and young oocyte groups. Alternative structures containing LC3B include intracellular vesicles that fuse with autophagosomes, such as lysosomes (to form autolysosomes) and endosomes (to form amphisomes) (Klionsky et al., 2014). To explore the possibility of these large puncta being amphisomes, immunocytochemistry experiments were employed to co-localize LC3B with the recognized endosome marker, early endosome antigen 1 (EEA1). The LC3B and EEA1 markers were revealed to highly co-localize within the large puncta (Figure 7A). The degree of this co-localization was determined using Pearson’s correlation coefficient illustrating equivalent distribution patterns in both young (*R* = 0.7686) and aged groups (*R* = 0.7618). To further confirm the presence of these two proteins within the same structures, a proximity ligation assay (PLA) was used. Notably, PLA produces a fluorescent signal wherever the epitopes of the target proteins reside within a maximum of 40 nm. In both young and aged oocytes, a strong PLA signal was displayed in a similar pattern to the co-localization experiments (Figure 7B). Combined, these data confirm that the large immunostained puncta (i.e., greater than 10 μm^2^) were likely to be amphisomes.

### Lysosome Number Decreases in Aged Mouse Oocytes

**FIGURE 8:**
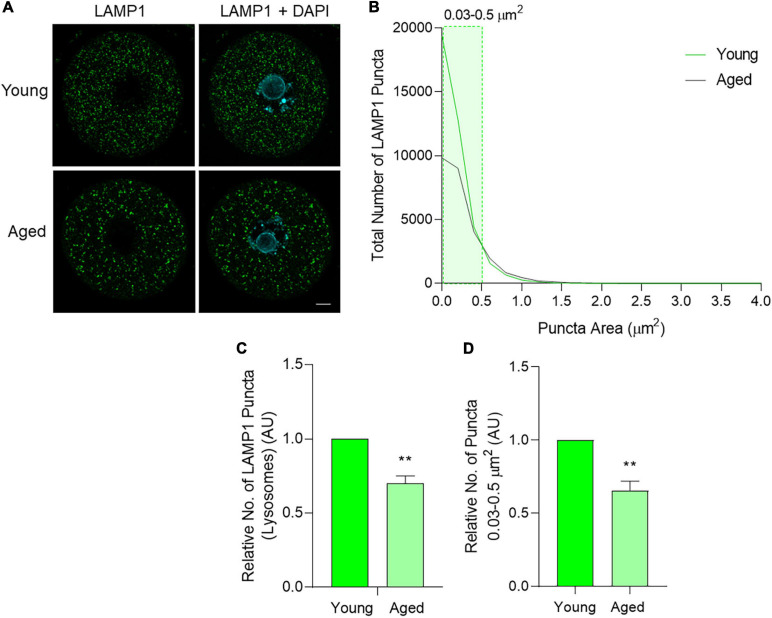
LAMP1 protein localization and puncta distribution in young and aged mouse oocytes. Oocytes from young (4–6 weeks old) and aged (12–14 months old) mice were fixed prior to labeling with anti-LAMP1 antibodies (green), nuclear counterstaining with DAPI (cyan), confocal imaging (60× objective), and puncta analysis. **(A)** Representative images illustrate the punctate cytoplasmic localization of LAMP1 in young and aged oocytes. Scale bar = 10 μm. **(B)** Images were assessed for the number and size of LAMP1 labeled puncta and these data were used to graph the size distribution of puncta in populations of young (green line) and aged (gray line) oocytes. The puncta size group that differed significantly in abundance between young and aged oocytes is highlighted in green shading. **(C)** Comparison of the total number of LAMP1 labeled puncta per oocyte in young and aged oocytes (normalized to young). **(D)** Comparison of LAMP1 labeled puncta sized 0.03–0.5 μm^2^ pertaining to lysosome area documented in literature (Ponsford et al., 2020; Trivedi et al., 2020) in young and aged oocytes (normalized to young). *N* = 4, **P ≤ 0.01.

Subsequently, the third macroautophagy pathway marker, lysosomal-associated membrane protein 1 (LAMP1), was also assessed through immunocytochemistry. The staining of LAMP1 displayed a dispersed, punctate distribution, reflective of lysosomal staining within a cell (Figure 8A). As with BECN1 and LC3B, the total distribution of the puncta number and area was first assessed. While lysosome size varies under physiological conditions, literature pertaining to somatic cells suggests lysosomes usually occupy a size range of between 0.03 and 0.5 μm^2^ (Ponsford et al., 2020; Trivedi et al., 2020). In the present study, a large number of LAMP1 puncta were observed within this range (Figure 7B shaded area), and the number of puncta decreased as area increased (Figure 8B). Notably, lysosomes can increase in size due to fusion with endosomes, phagosomes, or autophagosomes, giving rise to structures that exceed the 0.03–0.5 μm^2^ range (Luzio et al., 2007). This shift in size may be reflected in our data set with fewer LAMP1 labeled puncta detected up to 4 μm^2^ in area (Figure 8B). Overall, there were clear differences when comparing the size distribution of lysosomes in young and aged oocytes, contributing to a 30% decrease in lysosomes in aged oocytes (*p* = 0.01) (Figure 8C). Observing specific areas of interest within the size distribution, the total decrease in lysosomes was primarily attributed to a decrease in lysosomes ranging from 0.03 to 0.5 μm^2^ in aged oocytes (*p* = 0.01) (Figure 8D).

### Lysosomal Inhibition Promotes the Formation of Large Amphisomes in Mouse Oocytes

**FIGURE 9:**
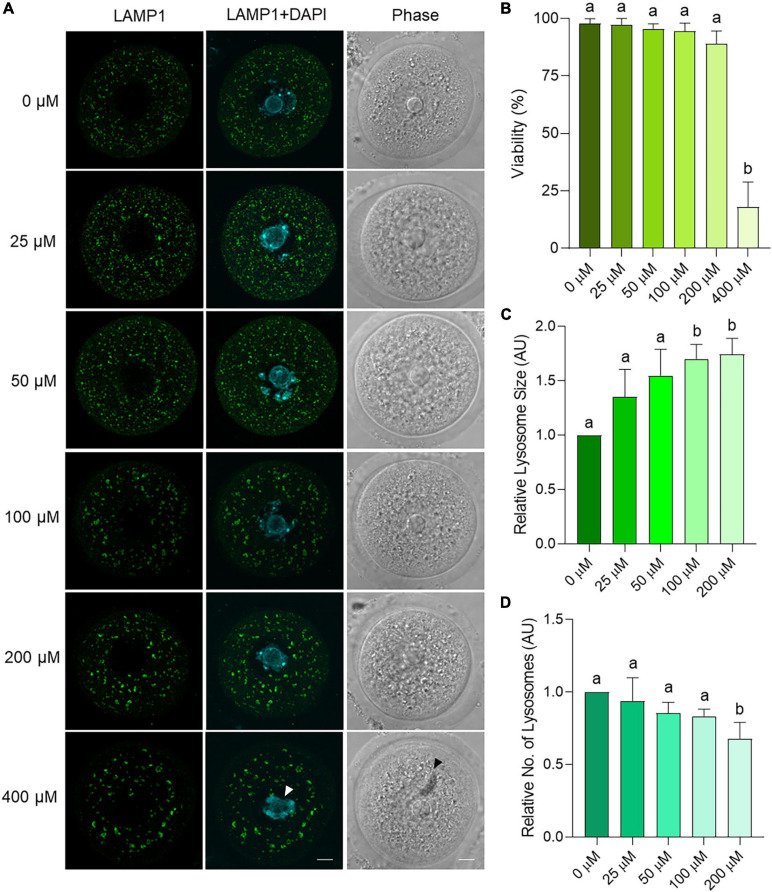
Dose response trial using chloroquine on young mouse oocytes to assess effects on lysosome parameters. Oocytes from young (4–6 weeks old) mice were treated for 6 h with varying concentrations of chloroquine before fixation and labeling with anti-LAMP1 antibodies (green), nuclear counterstaining with DAPI (cyan), confocal imaging (60× objective), and puncta analysis. Scale bar = 10 μm. **(A)** Representative images illustrate the changes in size and number of LAMP1 labeled puncta within oocytes treated with 0, 25, 50, 100, 200, and 400 μM chloroquine. White arrow indicates reduced chromatin condensation and black arrow indicates cytoplasmic inclusion. **(B)** Viability of oocytes as the percentage of live, morphologically normal oocytes in each treatment group. **(C)** Average area and **(D)** number of lysosomes (LAMP1-staining puncta) as determined through puncta analysis. Data is normalized to the untreated control. Statistical significance (*P* < 0.05) between groups is indicated by different letters, *n* = 3 for all groups except 0 and 100 μM with *n* = 6. Statistics not performed on 400 μM due to low viability.

To explore the mechanism(s) by which an accumulation of amphisomes may occur in aged oocytes, an *in vitro* model was established in which oocytes from young mice were incubated with chloroquine. Chloroquine is an inhibitor of lysosomal activity that effectively prevents the fusion of lysosomes with autophagosomes and increases lysosomal area (Mauthe et al., 2018). Initially, effective chloroquine concentrations were titrated to encompass doses between 25 and 400 μM that were previously used in somatic cell and oocyte studies (Pellegrini et al., 2014; Jia et al., 2018; Mauthe et al., 2018; Mihalas et al., 2018; Miao et al., 2019). Specifically, oocytes were co-incubated with chloroquine (25–400 μM) for 6 h, after which LAMP1 immunolocalization was assessed to measure lysosomal area and number. Importantly, all treatment groups displayed comparable viability (>89%) and cellular morphology to the population of untreated control oocytes except for those exposed to the highest chloroquine dose of 400 μM (*p* < 0.0001) (Figure 9B). This dose resulted in abnormal oocyte morphology, reduced DNA condensation, and the formation of large cytoplasmic inclusions indicating significant toxicity and was excluded from further analysis (Figure 9A arrows). All other concentrations of chloroquine yielded a dose-dependent increase in lysosome area, with this response achieving statistical significance at concentrations of 100 and 200 μM chloroquine (*p* = 0.0048 and 0.0150, respectively) (Figures 9A,C). Lysosomal number also significantly decreased at 200 μM (*p* = 0.0257) (Figure 9D). Due to the 200 μM chloroquine treatment having the highest efficacy, this concentration was selected for use in further experiments.

**FIGURE 10:**
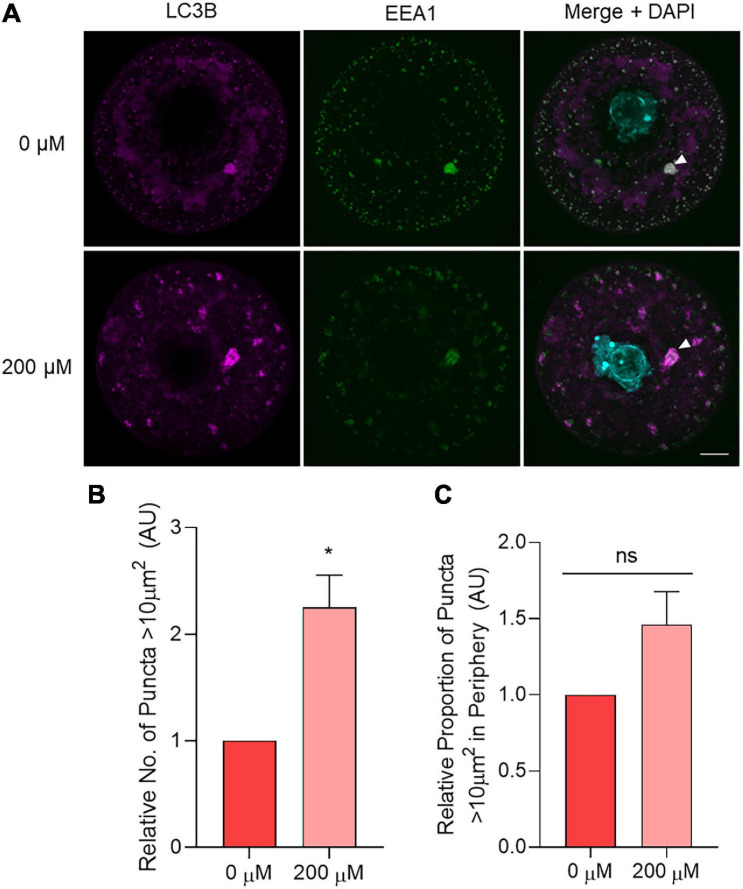
The effect of chemical lysosomal inhibition using chloroquine on LC3B and EEA1 immunolocalization in young oocytes. Oocytes from young (4–6 weeks old) mice were treated for 6 h with 0 μM (control) or 200 μM chloroquine before fixation and labeling with anti-LC3B antibodies (pink), anti-EEA1 antibodies (green), nuclear counterstaining with DAPI (cyan), confocal imaging (60× objective), puncta analysis, and peripheral analysis using Fiji. **(A)** Representative images demonstrate staining of LC3B and EEA1 in control and treated oocytes where white arrow points to example of large puncta. Scale bar = 10 μm. **(B)** Comparison of the number of LC3B labeled puncta >10 μm^2^ in control and treated oocytes (normalized to untreated control). **(C)** Comparison of the proportion of LC3B labeled puncta >10 μm^2^ that resided in the periphery (outermost 10 μm) of the oocyte (normalized to untreated control). *N* = 3, *P < 0.05, ns, not significant.

**FIGURE 11:**
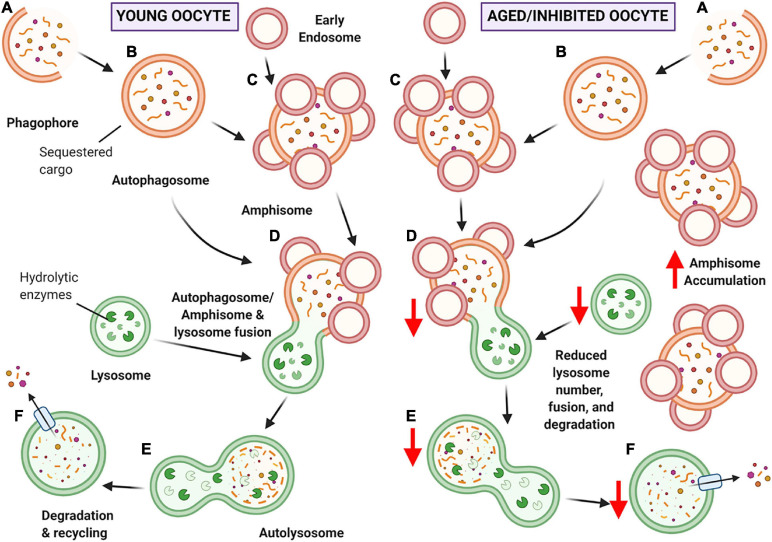
Schematic of hypothesized ‘bottleneck model’ of macroautophagy in aging or chloroquine treated oocytes. **(A)** Macroautophagy begins with the formation of the phagophore, and as it continues to grow and sequester cargo, it forms a completed double-membraned vesicle termed the autophagosome, encompassing the cargo to be degraded **(B)**. Based on our data, the autophagosome may then fuse with early endosomes to create amphisomes **(C)**. The autophagosome or amphisome can then fuse with the acidic lysosome containing hydrolytic enzymes **(D)** to form an autolysosome **(E)** in which the encapsulated cargo is broken down into its basic building blocks. Lastly, the broken down cargo is extruded back into the cell cytoplasm for recycling **(F)** (Feng et al., 2014; Klionsky et al., 2016). In young oocytes (left-hand panel) this process is hypothesized to occur normally. In aged or chloroquine treated oocytes (right-hand panel), the availability of lysosomes is decreased, resulting in less lysosomal fusion with autophagic vesicles and ultimately reduced protein degradation. Due to the bottleneck created by the reduced lysosomal availability (due to decreased numbers in aging or loss of functionality with inhibition) an accumulation of large amphisomes is observed. Created with BioRender.com.

After optimizing this lysosomal inhibition strategy, oocytes treated with 200 μM chloroquine for 6 h were assessed for immunolabeling patterns of LC3B, EEA1, and LAMP1. LAMP1 was used to ensure the consistency of inhibitor-induced lysosomal dilation across replicates (Supplementary Figure 4). Assessing LC3B and EEA1 staining, a punctate distribution featuring irregularly shaped, less rounded puncta (potential amphisomes) was observed in chloroquine treated oocytes (Figure 10A). Similarly to aged oocytes, a significant increase in the number large puncta greater than 10 μm^2^ was evident in the 200 μM chloroquine treatment group (Figure 10B). Following this, the colocalization of LC3B and EEA1 within these large puncta (i.e., area > 10 μm^2^) was quantified. This colocalization analysis revealed a highly significant (*p* = 0.0016), 38% reduction in the dual-labeling of these markers in chloroquine challenged oocytes compared to the untreated control (*R* = 0.515 and 0.829, respectively). While the localization of the large puncta tended to increase in the periphery of the treated cells compared to controls cells, this did not yield a significant difference (*p* = 0.0987) (Figure 10C). Combined, these results provide a mechanistic link between the compromise of lysosomal function and the accumulation of amphisomes characteristic of naturally aged oocytes (Figure 11).

## Discussion

Aging oocytes experience a decrease in quality, eventuating in poor developmental outcomes for the subsequent embryo (te Velde and Pearson, 2002; Doherty and Pal, 2011). Despite continuing efforts to pinpoint critical mechanisms involved in the loss of oocyte quality, there has been minimal progression in fertility treatment and preservation for women of advanced reproductive age. Macroautophagy is an essential protein degradation pathway that decreases in efficiency in aging somatic cells (Terman, 1995; Vilchez et al., 2014; Peters et al., 2020). However, few studies have sought to investigate the contribution of this pathway to the age-dependent deterioration of oocyte quality. In recognizing this knowledge gap, we have used comprehensive image analysis techniques to demonstrate that the localization of crucial macroautophagy pathway markers is modulated with age in the pre-ovulatory mouse oocyte. Moreover, we established a novel short-term treatment model that recapitulates the hallmark features of macroautophagy found in aged oocytes, revealing decreased lysosome abundance as the mechanism underpinning these changes.

Upon comparison of the three autophagy pathway members *Becn1/*BECN1, *Map1lc3*/LC3B, and *Lamp1/*LAMP1, no gross changes in gene or protein expression were detected between young and aged oocyte groups. This indicated that the overall expression of autophagy pathway members remains consistent in pre-ovulatory oocytes throughout the aging process. LC3B proteins exist in two forms within the cytoplasm; LC3B-I, which exists as free protein in the cytoplasm, and LC3B-II, the lipidated protein within the autophagosome membrane (Klionsky et al., 2016). In our study, only LC3B-II was detected in oocytes indicating that the LC3B within the oocyte was bound within autophagosomes. By contrast, both forms of LC3B were detected in whole ovarian tissue lysates. One possible explanation for this is that LC3B-I could be expressed in the somatic cells surrounding the oocyte, whereby LC3B-I is converted to LC3B-II and then transported to the oocyte. In line with this, it is known that oocytes possess transzonal projections enabling the surrounding granulosa cells to transport molecules to the oocyte cytoplasm to aid function and growth (Gilchrist et al., 2008). This generally low abundance of LC3B may also be linked to its relative gene expression, where we found *Map1lc3* exhibited significantly lower expression than that of *Becn1* and *Lamp1*. Additionally, the low abundance and expression of *Map1lc3*/LC3B could be indicative of an autoregulatory ‘brake’ mechanism. This autoregulatory ‘brake’ can occur when upstream members of the pathway, including mammalian target of rapamycin (mTOR) and death-associated protein 1 (DAP1), are activated to prevent overactivation of autophagy (Koren et al., 2010). This mechanism has been demonstrated to occur under conditions of nutrient deprivation and rapamycin treatment (pharmacological activation) (Koren et al., 2010); however, it is yet to be determined if this mechanism occurs in the oocyte.

BECN1 is a component of the phosphoinositide 3-kinase (PI3K) nucleation complex involved in activating macroautophagy and is a marker commonly used to monitor autophagy (Klionsky et al., 2016). However, there is increasing evidence that BECN1 is also involved in non-autophagy-related pathways (Martinet et al., 2013; Myung Park et al., 2014; Xu et al., 2017). Immunocytochemistry experiments within this study demonstrated high variability of BECN1 puncta localization in the cytoplasm and nucleus within all oocytes regardless of their age group. This nuclear localization may be explained by the involvement of *Becn1* in DNA double-stranded break repair pathways. This involvement has been demonstrated in studies assessing radiation-induced DNA damage where *Becn1* has been implicated in regulating DNA double-strand break repair mechanisms (Myung Park et al., 2014; Xu et al., 2017). Due to this association of BECN1 with non-autophagy-related DNA double-strand break repair pathways that become dysfunctional with aging in the oocyte (Titus et al., 2013; Turan and Oktay, 2020), this commonly used marker was not used in subsequent experiments.

In contrast, LC3B displayed clear punctate cytoplasmic localization with a differing expression between young and aged oocytes. Subsequently, LC3B, in combination with LAMP1, was employed as a primary marker of macroautophagy in the remainder of this study. LC3B is a reliable and widely used maker of autophagy due to its role as a component of the autophagosome membrane (Klionsky et al., 2016). It has previously been suggested that a decline in autophagy efficiency due to declined autophagosome numbers in aging somatic cells arises from decreased formation and elimination of autophagosomes (Cuervo et al., 2005; Wilhelm and Richly, 2018). This decrease in the formation of autophagosomes is reflected in our dataset, whereby a reduction in the number of autophagosomes (0.196–1.767 μm^2^ category) and phagophores (<0.196 μm^2^ category) was observed in aged oocytes compared to their young counterparts. Accompanying this, an increase in large puncta greater than 10 μm^2^ was evident. These large puncta were subsequently identified as amphisomes through the co-localization of LC3B and EEA1, an early endosome marker. The accumulation of these large amphisomes, accompanied by a concomitant decrease in autophagosomes, was a key finding within this study. Amphisome accumulation has also been observed in other cell types, including aging neurons in Alzheimer’s disease and hepatocytes exposed to leupeptin, a protease inhibitor that prevents lysosomal protein degradation (Berg et al., 1998; Tammineni and Cai, 2017).

The accumulated amphisomes documented in our study were substantially larger than typical autophagosomes (0.196–1.767 μm^2^) and endosomes (0.071–0.785 μm^2^) (Mizushima et al., 2002; Skjeldal et al., 2012). Amphisomes can comprise early and late endosomes fused with autophagosomes and can undergo multiple independent fusions with other endosomes (Berg et al., 1998). Additionally, defects in the endocytic pathway can cause early endosome enlargement (Kaur and Lakkaraju, 2018). By way of example, Ras-associated binding protein RAB5, a GTPase that controls the fusogenic properties of early endosomes, can be altered to a mutant form known as RAB5(Q79L). This mutant RAB5(Q79L) causes endosomes to retain early endosome markers as they mature and become ‘giant endosomes’ (Wegner et al., 2010). These structures retain the ability to fuse with other such endosomes, leading to a dramatic expansion in vesicle size. In a similar study, the overexpression of Rab5 led to an increased endosome size of up to 10 μm in diameter (Skjeldal et al., 2012). In this way, these mechanisms provide viable explanations for how oocyte amphisomes may reach the large sizes documented here. Overall, our observations are the first of amphisomes in aging oocytes that are larger than those observed naturally occurring in other cells, indicating that oocyte aging presents a challenge to the normal autophagic response.

It is also noteworthy that large amphisomes were typically localized to the periphery of aged oocytes. In seeking an explanation for this observation, it is known that the movement of intracellular vesicles, including amphisomes, autophagosomes, and lysosomes is reliant on the cytoskeleton network (Kamal and Goldstein, 2000; Nakamura and Yoshimori, 2017). The directional movement of vesicles is determined by various microtubule motor proteins that move toward the periphery (e.g., kinesin superfamily proteins KIF5 and KIF3) or perinuclear region (e.g., KIFC1, 2, and 3) depending on the plus or minus orientation of the microtubules to which they attach (Hirokawa et al., 2009; Camlin et al., 2017a; Muralidharan and Baas, 2019). A recent study assessing the microtubule motor control of early endosomes in human endothelial cells demonstrated that simultaneous silencing of KIFC1 and stromal interaction molecule 1 (STIM1) resulted in more peripherally distributed EEA1 positive early endosomes (Villari et al., 2020). Aligning with this, *Kifc1* gene and KIFC1 protein expression decreases in the aged oocyte, with functional siRNA-mediated knockdown and pharmacological inhibition of *Kifc1*/KIFC1 in young oocytes recapitulating hallmarks of poor oocyte quality such as increased aneuploidy rates (Mihalas et al., 2019). Taken together, these findings provide a putative explanation for the peripheral distribution of amphisomes noted in our study and provide the impetus to investigate the role of KIFC1 and other KIF protein members in facilitating macroautophagy in the aging oocyte.

Here, it was hypothesized that the observed increase in amphisomes in aged oocytes was attributed to decreased rates of lysosomal clearance. Consistent with this notion, the assessment of LAMP1 puncta as a proxy for lysosomes revealed a pronounced decrease in lysosome number among aged oocytes. It should be noted that this decrease was restricted to puncta within the size range pertaining to lysosomes (0.03–0.5 μm^2^). By contrast, larger LAMP1 labeled structures of up to 4 μm^2^ were present in both young and aged oocytes. It is possible that the persistence of these larger LAMP1-containing structures may account for the detection of equivalent amounts of LAMP1 protein (as assessed via densitometric analysis of LAMP1 immunoblots) across young and aged oocyte groups. Although the cause of the age-related decrease in lysosomes is yet to be established, we postulate at least two possible mechanisms by which this phenomenon may occur. Firstly, oxidative insults arising under conditions of cellular oxidative stress may compromise the integrity of the lysosomal membranes and promote lysosomal rupture (Brunk et al., 1997). In agreement with this model, chronic oxidative stress is a well-established driver of oocyte aging (Goud et al., 2008). If this were to hold true, the rupture of lysosomes could exacerbate oxidative damage within oocytes and contribute to the presentation of additional, functionally harmful hallmarks of aging such as lipofuscin accumulation (Brunk et al., 1997). An alternative explanation for decreased lysosomal number lies upstream in the biogenesis of these organelles. In this context, several lysosome biogenesis-related pathways share transcriptional control under the transcription factor EB (TFEB) (Saftig and Klumperman, 2009; Bajaj et al., 2019). It follows that TFEB dysregulation contributes to the pathogenesis of lysosomal storage diseases and neurodegenerative diseases associated with aging (Sardiello, 2016; Bajaj et al., 2019; Cortes and La Spada, 2019). This age-associated dysregulation of TFEB in long-lived neuronal cells can disrupt lysosome biogenesis and reduce lysosome number (Cortes and La Spada, 2019); while yet to be elucidated, this mechanism could be a potential contributor to reduced lysosome number in oocytes. Irrespective of the mechanism, the decreased prevalence of lysosomes within aged oocytes could conceivably create a bottleneck effect leading to an upstream accumulation of amphisomes within these cells. Ultimately, these observations may reflect a reduction in the efficiency of protein degradation and hence a delay in the removal of damaged or dysfunctional protein cargo contained within the amphisomes. This could compromise oocyte maturation with potential repercussions for post-fertilization zygotic development, a possibility that warrants further investigation.

In order to model decreased lysosome availability, the lysosomal inhibitor chloroquine was used to prevent the fusion of autophagosomes and lysosomes (Mauthe et al., 2018), mimicking the bottleneck effect described above. Notably, oocytes treated with 200 μM chloroquine displayed an increased number of amphisomes greater than 10 μm^2^, revealing that amphisome accumulation in the aged oocyte is likely linked to reduced lysosome availability. In the presence of chloroquine, amphisome morphology was characterized by irregularly shaped structures and a reduced abundance of embedded EEA1. These features varied substantially from the uniform amphisomes present in untreated control oocytes. In addition to lysosomal accumulation, chloroquine can also accrue within acidic endosomal organelles, leading to perturbation of their acidity, increased osmotic pressure, and eventual membrane rupture; responses that have been exploited in the context of drug delivery and release within target cells (Yu et al., 2016; Hajimolaali et al., 2021). Given this insight, it is possible that chloroquine action within the oocyte may be attributed to a compromise of endosome integrity, accounting for the irregular amphisome morphology witnessed after chloroquine challenge. While chloroquine treatment successfully modeled the physiological mechanism(s) responsible for amphisome accumulation in aged oocytes, it is unlikely to mirror all aspects of macroautophagy compromise due to the multifaceted, complex nature of aging. Nevertheless, this treatment regimen did replicate a key feature of macroautophagy in the aging oocyte by preventing damaged and dysfunctional proteins from being cleared from the cells. In this way, amphisome accumulation caused by decreased lysosomal efficiency could be a symptom of dysfunctional autophagy contributing to the age-dependent deterioration of oocyte quality. This treatment regimen may prove valuable for future investigation into the mechanisms underlying altered autophagy pathway responses during female reproductive aging. Specifically, it could shed light on the behavior of upstream members of the macroautophagy pathway and aid in assessing the effects of reduced lysosomal efficiency and amphisome accumulation during oocyte development and maturation. Ultimately these investigations will contribute to the development of novel prophylactic drugs that could be used to prevent lysosomal decline throughout oocyte aging and hence maintain oocyte quality.

In summary, this study is the first to utilize image-based analysis to discern signature changes occurring in the macroautophagy pathway in maternally aged oocytes, revealing fundamental changes that could contribute to reduced oocyte quality. Among the key changes documented were age-dependent reductions in the number of autophagosomes and lysosomes and a reciprocal accumulation of amphisomes, all of which have significant functional implications for oocyte proteostasis. Furthermore, we have demonstrated that amphisome accumulation can be induced via the inhibition of lysosome activity in oocytes, providing a robust model of macroautophagy degeneration in aging oocytes. Collectively, these data demonstrate a role for macroautophagy in the maintenance of pre-ovulatory oocyte health and provide the impetus to focus on therapeutic interventions that modulate this pathway to promote oocyte longevity and combat the age-related decline in female fertility.

## Data Availability Statement

The original contributions generated for this study are included in the article/Supplementary Material, further inquiries can be directed to the corresponding author.

## Ethics Statement

The animal study was reviewed and approved by University of Newcastle Animal Care & Ethics Committee.

## Author Contributions

AP and JS contributed substantially to conception and design, acquisition of data, analysis and interpretation of data, drafting the article and revising it critically for important intellectual content, and final approval of the version to be published. SC, SR, EB, and BN contributed to acquisition of data, analysis and interpretation of data, critical revision of article for important intellectual content, and final approval of the version to be published. EM contributed to critical revision of article for important intellectual content and final approval of the version to be published. All authors contributed to the article and approved the submitted version.

## Conflict of Interest

The authors declare that the research was conducted in the absence of any commercial or financial relationships that could be construed as a potential conflict of interest.

## Footnotes

1 The use of the term ‘women’ here refers to people assigned female at birth and possessing a female reproductive system. The authors acknowledge that not all people who have female reproductive systems are women.

## Abbreviations

*Becn1*/BECN1: Beclin-1
DAP1: death associated protein 1
EEA1: early endosome antigen 1
GAPDH: anti-glyceraldehyde 3-phosphate dehydrogenase
KIF: kinesin super family protein
*Lamp1*/LAMP1: lysosomal associated membrane protein 1
LC3B: microtubule associated protein 1 light chain 3B
*Map1lc3/L3c*: microtubule associated protein 1 light chain 3
mTOR: mammalian target of rapamycin
PI3K: phosphoinositide 3-kinase
*Ppia*: peptidylprolyl isomerase A
RAB: Ras-associated binding protein
STIM1: stromal interaction molecule 1
TFEB: transcription factor EB
ULK: unc-51-like kinase 1.
