# Supplementary material for: The Impact of Aging on Macroautophagy in the Pre-ovulatory Mouse Oocyte
Source: Front Cell Dev Biol. 2021 Jun 29;9:691826. doi: 10.3389/fcell.2021.691826 (PMC8277196; doi:10.3389/fcell.2021.691826)
Supplement: Supplementary file 1 [file Data_Sheet_1.DOCX]

Supplementary Material

## Supplementary Tables

**Supplementary Table 1: Primary antibody details and concentrations**

| **Protein target of antibody** | **Manufacturer** | **Catalogue Number** | **Initial concentration** | **Application and dilution** | **RRID** |
| --- | --- | --- | --- | --- | --- |
| **BECN1** | Novus Biologicals, Littleton, CO, USA | NB500-249 | 1 mg/ml | Immunoblot (1:2000) and immunocytochemistry (1:200) | AB_10001277 |
| **LC3B** | Abcam, Cambridge, UK | ab192890 | 0.908 mg/ml | Immunoblot (1:1000) | AB_2827794 |
| **LC3B** | Abcam, Cambridge, UK | ab48394 | 1 mg/ml | Immunocytochemistry and proximity ligation assay (1:100) | AB_881433 |
| **LAMP1** | Abcam, Cambridge, UK | ab24170 | 1 mg/ml | Immunoblot (1:1000) and immunocytochemistry (1:100) | AB_775978 |
| **EEA1** | BD Biosciences, Franklin Lakes, NJ, USA | 610456 | 250 µg/ml | Immunocytochemistry and proximity ligation assay (1:25) | AB_397829 |
| **GAPDH** | Merck, Darmstadt, Germany | G9545 | 1 mg/ml | Immunoblot (1:5000) | AB_796208 |
| **PIWIL1** | Abcam, Cambridge, UK | ab12337 | 1 mg/ml | Proximity ligation assay (1:100) | AB_470241 |
| **α-tubulin** | Thermo Fisher Scientific, Waltham, MA, USA | A11126 | Not specified | Proximity ligation assay (1:400) | AB_2534135 |
| **β-tubulin** | Abcam, Cambridge, UK | ab6046 | 1 mg/ml | Proximity ligation assay (1:200) | AB_2210370 |

## Supplementary Figures

**
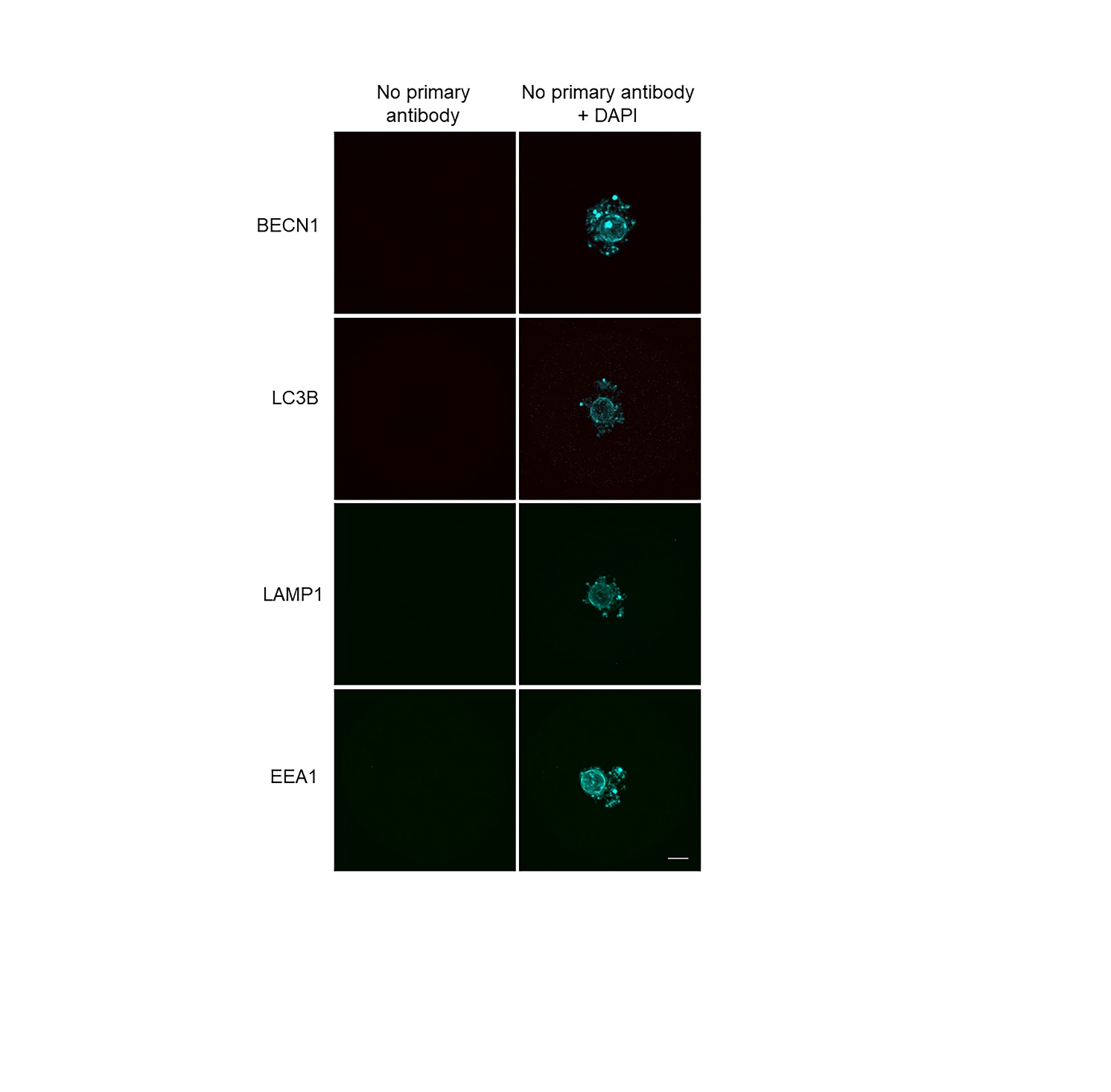
**

**Supplementary Figure 1: Control oocytes that underwent immunocytochemistry omitting primary antibodies alongside BECN1, LC3B, LAMP1, and EEA1 stained oocytes**. Oocytes from young (4-6 weeks old) and aged (12-14 months old) mice were fixed prior to sequential immunocytochemistry where primary antibodies were substituted for anti-body diluent, nuclear counterstaining with DAPI (cyan), and confocal imaging (60× objective). Representative images are presented to illustrate the lack of non-specific staining in oocytes due to immunocytochemical procedure. Scale bar = 10µm.


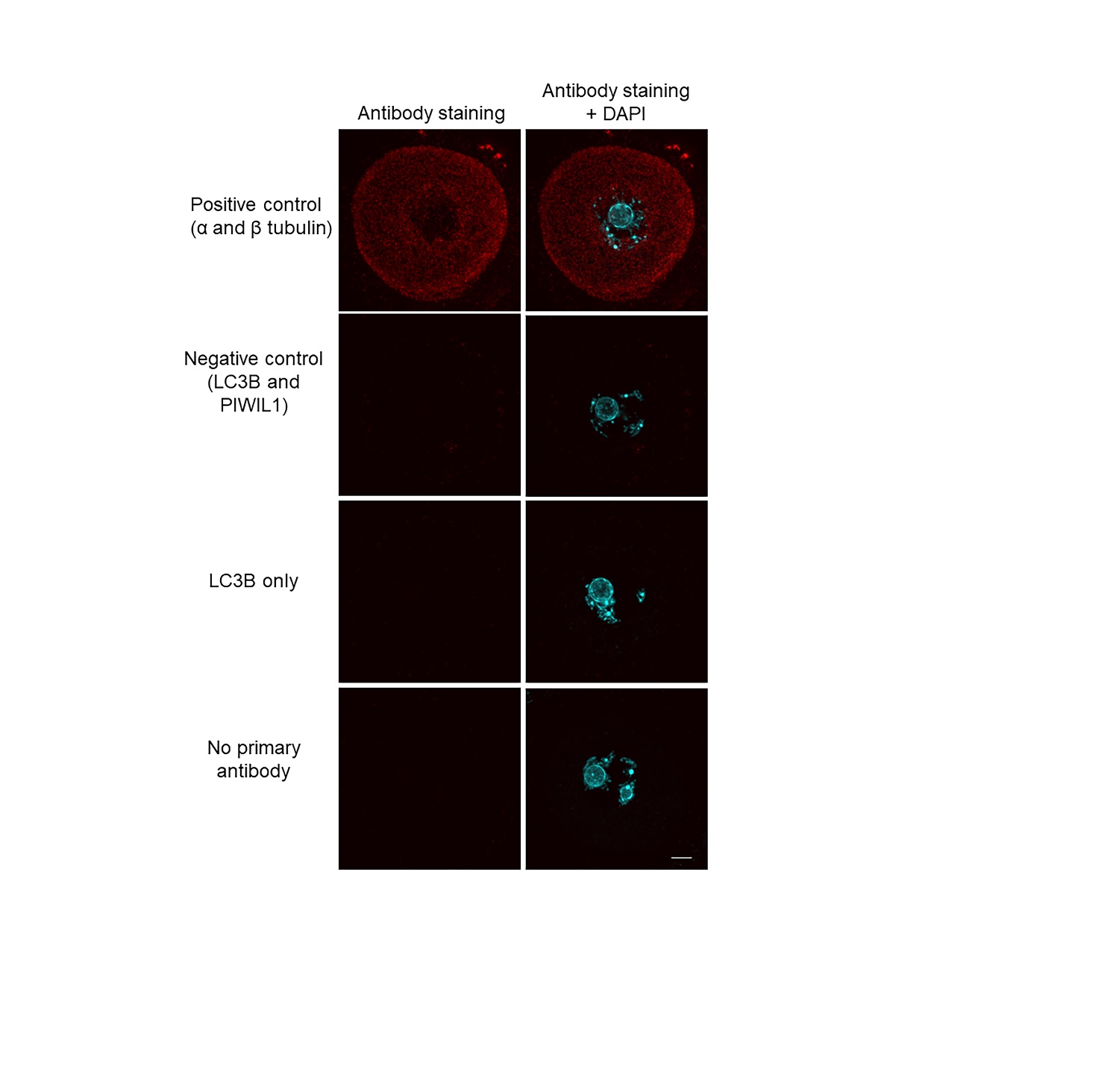


**Supplementary Figure 2: Positive control, negative control, single antibody control, and no primary antibody control for proximity ligation assay.** Fixed young (4-6 weeks old) and aged (12-14 months old) oocytes underwent a proximity ligation assay for LC3B and EEA1 alongside various controls and were imaged through confocal microscopy (60× objective). Representative images are presented to illustrate the positive control using antibodies for proteins known to interact or be within 40 nm (α-tubulin and β-tubulin), negative control using antibodies for proteins unlikely to interact (LC3B and PIWIL1), single antibody control using the anti-LC3B antibody, and control omitting all primary antibodies. Scale bar = 10µm.

**
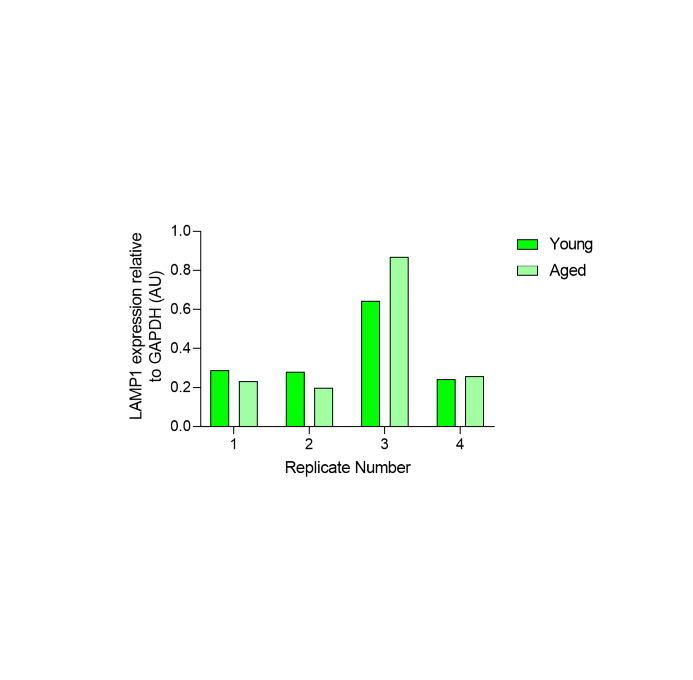
**

**Supplementary Figure 3: LAMP1 protein expression (measured through densitometric analysis) for each biological replicate in young and aged oocytes.** Protein lysates prepared from oocytes of young (4-6 weeks old) and aged (12-14 months old) mice were subjected to immunoblotting with anti-LAMP1 (120kDa) antibodies. Following detection of the target antigens, the blots were stripped and reprobed for the housekeeping protein GAPDH (35kDa) to ensure equivalent protein loading in each lane. Densitometric analysis was performed on labeled bands corresponding to the predicted molecular weight of target proteins to enable protein abundance to be determined relative to that of GAPDH. Densitometric data is presented relative to GAPDH (n = 4), non-normalized to young group.

**
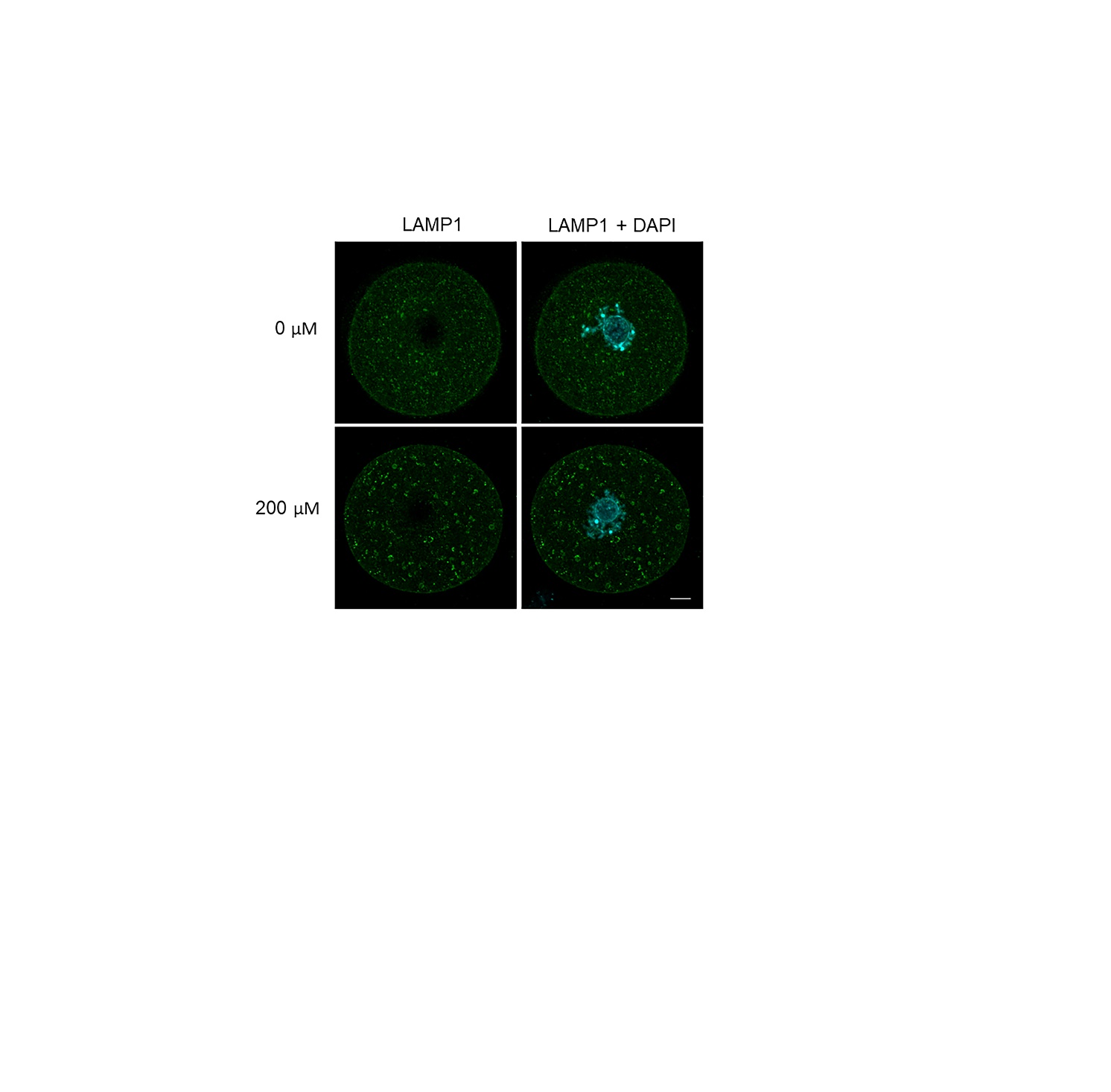
**

**Supplementary Figure 4: LAMP1 immunocytochemistry showing lysosomal dilation in oocytes treated with 200 µM chloroquine.** Oocytes from young (4-6 weeks old) and aged (12-14 months old) mice were fixed prior to sequential labeling with anti-LAMP1 antibody (green), nuclear counterstaining with DAPI (cyan), and confocal imaging (60× objective). Representative images are presented to illustrate the lysosomal dilation that occurred as a result of 200 µM chloroquine treatment alongside staining for LC3B and EEA1 presented in the main text. Scale bar = 10µm.
